# Impact of hemoperfusion with polymyxin B added to hemofiltration in patients with endotoxic shock: a case–control study

**DOI:** 10.1186/s13613-018-0465-8

**Published:** 2018-12-07

**Authors:** Ana Navas, Ricard Ferrer, Maria Luisa Martínez, Gemma Gomà, Gisela Gili, Jordi Masip, David Suárez, Antonio Artigas

**Affiliations:** 1 Critical Care Center, Parc Tauli Hospital Universitari, Institut d’Investigació i Innovació Parc Taulí I3PT, Universitat Autònoma de Barcelona, Sabadell, Spain; 2 CIBER Respiratory Diseases, Madrid, Spain; 3 Intensive Care Department, Vall d’Hebron University Hospital, Shock, Organ Dysfunction and Resuscitation Research Group, Vall d’Hebron Research Institute, Barcelona, Spain; 4 Department of Intensive Care, Hospital Universitari General de Catalunya, Barcelona, Spain; 5 Unitat de Suport a la Investigación Clínica, Vall d’Hebron Institut de Recerca, Barcelona, Spain; 6 Servei de Medicina Intensiva, Hospital Universitari Sant Joan de Reus, Tarragona, Spain; 7 Epidemiology and Assessment Unit, Fundació Parc Tauli, Universitat Autònoma de Barcelona, Sabadell, Spain

**Keywords:** Endotoxic shock, Hemoperfusion, Polymyxin B, Acute kidney injury, Hemofiltration

## Abstract

**Background:**

Septic shock is a leading cause of death in critical patients. In patients with gram-negative septic shock, hemoperfusion with polymyxin B aims to remove endotoxins from plasma. We analyzed the clinical and biological response to hemoperfusion in patients with septic shock and acute kidney injury.

**Methods:**

This prospective case–control study in the medical–surgical intensive care unit of a university hospital included consecutive adults patients with septic shock and suspected gram-negative bacteria infection with elevated plasma endotoxin activity (EAA > 0.6 EU/ml) and acute kidney injury requiring continuous renal replacement therapy (CRRT). At onset of septic shock, half underwent CRRT plus hemoperfusion with polymyxin B for two hours a day during two consecutive days (hemoperfusion group) and half received only CRRT (control group). We measured clinical, physiological, and biological parameters (EAA, C-reactive protein, procalcitonin, and cytokines) daily during the first 5 days.

**Results:**

We included 18 patients (male, 33%; mean age, 67.5; mean SOFA score, 11.3). Abdominal infections predominated (50% had peritonitis). At the beginning of CRRT, RIFLE classification was “failure” for 72% and “injury” for 28%. Baseline characteristics did not differ between groups. Patients in the hemoperfusion group required longer mechanical ventilation (12.4 vs. 9.4 days, *p* = 0.03) and CRRT (8.5 vs. 6 days, *p* = 0.01) than in the control group. Noradrenaline doses, lactate, procalcitonin, and C-reactive protein decreased in both groups. At day 5, EAA was significantly lower in the hemoperfusion group (0.58 EU/ml vs. 0.73 EU/ml in controls, *p *= 0.03). There were no significant differences between groups in other biomarkers or ICU mortality (33.3% in the treatment group vs. 44.4% in the control group, *p *= 0.5). No adverse effects of hemoperfusion were observed.

**Conclusions:**

Hemoperfusion with polymyxin B added to CRRT resulted in faster decrease in endotoxin levels, but we observed no improvements in clinical, physiological, or biological parameters.

**Electronic supplementary material:**

The online version of this article (10.1186/s13613-018-0465-8) contains supplementary material, which is available to authorized users.

## Background

Despite continuous improvements in the care of critical patients, septic shock is common and remains a leading cause of death [1]. Mortality rates for septic shock range from 28% to 50%, depending on the type of causal microorganism, source of infection, age, sex, comorbidities, severity of disease, and inflammatory response.

In septic shock caused by gram-negative bacteria, endotoxin activates the toll-like receptor 4 (TLR-4), generating an inflammatory response including an increase in proinflammatory cytokines and a coagulation cascade with increased neutrophils, endothelial and systemic damage, vasodilation, and increased interstitial permeability resulting in secondary edema at the tissue level [2].

Initial management and treatment of septic patients has improved since the publication of the Surviving Sepsis Campaign guidelines, which prioritize early diagnosis, early broad-range antibiotic administration, source control, and hemodynamic management [3]. In septic patients with acute kidney injury, continuous renal replacement techniques (CRRT) have been extensively studied with the rationale that extracting inflammatory mediators from patients’ plasma would be beneficial; however, attempts to correlate the dose or timing of CRRT with outcomes have failed [4, 5]. Most studies of CRRT in septic patients found decreased cytokines, but no improvement in survival [6, 7].

Hemoperfusion with polymyxin B (Toraymyxin^®^, Toray Industries) is a blood purification technique in which the patient’s plasma is filtered through a cartridge containing polyurethane and polystyrene-derivative fibers with polymyxin B, an antibiotic that has a high affinity for endotoxin. This adsorptive technique eliminates circulating endotoxin by covalent bonding (1:1) to polymyxin B [8]. Endotoxin concentrations higher than 500 pg/ml (> 0.6 EU/ml) in septic shock patients are associated with poor outcome [9–11].

Preclinical studies have shown that hemoperfusion with polymyxin B adsorbs endotoxin from circulating blood. Hemoperfusion with polymyxin B also decreases the major inflammatory cytokines and procalcitonin. However, various studies and meta-analyses have reported disparate results about the effects of hemoperfusion with polymyxin B on clinical outcomes [12–30].

We aimed to analyze the clinical, physiological, and biological effects of hemoperfusion with polymyxin B in patients with endotoxic shock and acute kidney injury treated with CRRT.

## Methods

### Study design

This prospective case–control study was conducted in a mixed ICU at a university hospital from January 1, 2008, to May 31, 2012. The Ethics Committee of the Hospital de Sabadell approved the protocol (CIR09065/CEAAH 2407), and all patients provided written informed consent.

We prospectively included consecutive adult patients with acute (< 48 h) septic shock (suspected bloodstream infection and need for vasoactive drugs) with an abdominal, biliary, or renal focus of infection, with acute kidney injury requiring CRRT (RIFLE score indicating injury or worse), and with elevated plasma endotoxin activity, defined as > 0.6 EU/ml on the Endotoxin Activity Assay (EAA™) (Spectral Diagnostics, Toronto, Canada) [9]. All patients received standard care according to the recommendations of the Surviving Sepsis Campaign [3]. All patients underwent hemofiltration with effluent flow rate 35 ml/kg/h through a double-lumen 13-F catheter and an AN-69 membrane through a PrismaFlex CRRT system (Baxter^®^); the system was anticoagulated with sodium heparin unless contraindicated, and bicarbonate-buffered solution was used as the replacement fluid. The first patients were assigned to the hemoperfusion group; in addition to hemofiltration, these patients underwent 2 h hemoperfusion with polymyxin B (Toraymyxin^®^, Toray Medical Co) on two consecutive days, starting within 24 h of ICU admission. The next consecutive patients who met the inclusion criteria were assigned to the control group; these patients underwent hemofiltration without hemoperfusion.

### Variables

We recorded the following variables: demographic characteristics (age and sex), severity (APACHE II), organ failure (SOFA scores), source of infection (peritonitis, biliary, or urinary tract infection), first antibiotic administered, technique used for infection source control, blood cultures, vasoactive drugs (doses and duration), mechanical ventilation, duration of CRRT, central venous oxygen saturation, and Pa0_2_/Fi0_2_ ratio.

All patients were monitored with a pulse index continuous cardiac output (PiCCO) system (Pulsion^®^), and the following hemodynamic variables were recorded: mean arterial pressure, vascular resistance, global end-diastolic volume, extravascular lung water index, pulmonary vascular permeability index, and global ejection fraction. Every 12 h during the first 2 days and then every 24 h during the next 3 days, blood samples were collected for standard biochemical analyses (pH, HCO_3_, serum creatinine, azotemia) and EAA determinations. Plasma samples were frozen for further analyses of various biomarkers (neutrophil gelatinase-associated lipocalin (NGAL), soluble urokinase-type plasminogen activator receptor (SuPAR), and cytokines (IL-6, IL-8, IL-10, TNF-α, IL-1β) (See supplementary material).

Patients were followed up until death or hospital discharge. Primary outcomes were variables related to the biological, physiological, and clinical effects of hemoperfusion with polymyxin B. Secondary outcomes were ICU and hospital lengths of stay and ICU and hospital mortality.

### Statistical analysis

Descriptive statistics included frequencies and percentages for categorical variables and means, standard deviations (SD), and confidence intervals (CI) for continuous variables. To compare categorical variables, we used the χ^2^ test or Fisher’s exact test, as appropriate. To compare continuous variables, we used Student’s *t* test or the Mann–Whitney *U* test, as appropriate. Statistical tests were two-tailed with significance defined as *p* < 0.05. We used SPSS, version 15.0 (SPSS, Chicago, Illinois), for all analyses.

## Results

A total of 18 patients were included: nine patients in the hemoperfusion group and nine in the control group. No adverse effects or coagulation of the circuit were observed in association with any of the 18 hemoperfusion treatments. Table 1 reports the demographic and baseline clinical characteristics of patients in the two groups and their overall outcomes. Demographic and baseline clinical characteristics were similar in the two groups. At the beginning of CRRT, 72% were classified as Failure according to RIFLE. The main source of sepsis was peritonitis, followed by biliary and urinary foci. Patients in the hemoperfusion group required longer mechanical ventilation (12.4 vs. 6.3 days, *p *= 0.03) and longer CRRT (8.5 vs. 3.5 days, *p *< 0.01). The mean ICU stay was 19.8 days, and the mean hospital stay was 36 days. Overall ICU mortality was 38.9% (33.3% in the hemoperfusion group vs. 44.4% in control group, *p *= 0.5); all in-hospital deaths occurred in the ICU. Mortality on day 2 (at end treatment) was 0% in the hemoperfusion group and 33% in the control group.

**Table 1:** Demographic and clinical characteristics of patients

	Global (*n* = 18)	Control patients (*n* = 9)	Toraymyxin treated patients (*n* = 9)	*p* value
Age: years, mean (SD)	67.5 (9.9)	66 (10)	69.1 (9.5)	0.52
Male: *n* (%)	6 (33.3)	2 (22)	4 (44)	0.31
Apache II: mean (SD)	20.67 (4.7)	21.2 (5.3)	20.1 (4.3)	0.63
SOFA baseline mean (SD)	11.3 (2.6)	11.6 (2.9)	11 (2.4)	0.6
Sepsis focus *n* (%)				
Peritonitis	9 (50)	4 (44)	4 (44)	0.4
Biliary tract	4 (23)	2 (22)	3 (33)	
Urinary tract	5 (27)	3 (33)	2 (22)	
RIFLE score (%)				
Injury	28	33	22	0.5
Failure	72	64	78	
Vasoactive drugs (days), mean (SD)	4.9 (3.8)	4.5 (3.4)	5.2 (4.3)	0.72
IMV (days), mean (SD)	9.4 (8.8)	6.3 (8)	12.4 (8.8)	0.03
CRRT (days), mean (SD)	6 (6)	3.5 (1.9)	8.5 (7.6)	0.01
ICU LOS (days), mean (SD)	19.8 (16.8)	14.7 (16)	24.9 (17)	0.21
Hospital LOS (days), mean (SD)	36 (31)	32 (34)	39.5 (29)	0.64
ICU mortality (%)	38.9	44.4	33.3	0.5
Hospital mortality (%)	38.9	44.4	33.3	0.5

*APACHE II* Acute Physiology and Chronic Health Evaluation II, *SD* standard deviation, *SOFA* Sequential Organ Failure Assessment, *IMV* invasive mechanical ventilation, *CRRT* continuous renal replacement therapy, *LOS* length of stay, *ICU* intensive care unit

Table 2 reports the source of sepsis, microbiology findings, and initial empiric antibiotic treatment in each patient. In accordance with the ICU’s protocol, all patients were treated with appropriate broad-spectrum antibiotics within 3 h of the diagnosis of septic shock, and antibiotic treatment was de-escalated according to the microbiology results. All patients underwent source control within 6 h of septic shock diagnosis. Stress-dose steroids (hydrocortisone, 100 mg every 8 h) were administered to 94%. All but one of the patients underwent invasive mechanical ventilation.

**Table 2:** Type of infection, microbiology findings, and initial empiric antibiotic treatment

	Sepsis focus	Microbiology findings	Empiric antibiotic treatment
Control patients			
Patient 1	Urinary tract	Negative	Meropenem plus vancomycin plus caspofungin
Patient 2	Urinary tract	*Escherichia coli*	Piperacillin–tazobactam
Patient 3	Urinary tract	*Escherichia coli*	Meropenem
Patient 4	Biliary tract	*Escherichia coli*/*Enterococcus faecalis*, *Enterococcus faecium*	Meropenem plus vancomycin
Patient 5	Peritonitis	*Escherichia coli*	Meropenem plus vancomycin
Patient 6	Peritonitis	Mixed flora	Meropenem plus amikacin
Patient 7	Biliary tract	*Escherichia coli*	Meropenem
Patient 8	Peritonitis	Negative	Meropenem
Patient 9	Peritonitis	*Enterococcus faecium*, *Candida albicans*, *Candida tropicalis*	Meropenem plus vancomycin plus anidulafungin
Toraymyxin treated patients			
Patient 1	Urinary tract	*Escherichia coli*	Meropenem plus vancomycin
Patient 2	Peritonitis	*Escherichia coli*/*Enterococcus faecium*/*Bacteroides fragilis*	Piperacillin–tazobactam plus fluconazole
Patient 3	Biliary tract	*Escherichia coli*/*Streptococcus anginosus*	Piperacillin–tazobactam plus fluconazole
Patient 4	Peritonitis	*Escherichia coli*	Piperacillin–tazobactam plus fluconazole
Patient 5	Biliary tract	*Escherichia coli*	Meropenem
Patient 6	Peritonitis	Mixed flora	Piperacillin–tazobactam
Patient 7	Peritonitis	Negative	Piperacillin–tazobactam
Patient 8	Urinary tract	*Escherichia coli*	Meropenem
Patient 9	Peritonitis	Mixed flora	Piperacillin–tazobactam

Table 3 reports the evolution of respiratory, hemodynamic, and renal parameters in the two groups over the first 5 days. Uremia, creatinine, and doses of noradrenaline decreased significantly compared to the baseline, but the decrease did not differ between groups. Changes in score SOFA between day 1 and day 5 did not differ between groups.

**Table 3:** Clinical results

Patients in hemoperfusion group/control group	Day 1 (pretreatment) (*n* = 18) 9/9	Day 2 (posttreatment) (*n* = 16) 9/7	Day 3 (*n* = 15) 9/6	Day 4 (*n* = 15) 9/6	Day 5 (*n* = 15) 9/6
Respiratory data, mean (SD)					
Pa02/Fi02 ratio					
Hemoperfusion group	260 (75)	260 (110)	242 (92)	222 (61)	251 (78)
Control group	209 (117)	218 (114)	247 (45)	215 (37)	185 (54)
HC03 (mmHg)					
Hemoperfusion group	17.4 (2.8)	22 (3)	25.4 (4.8)	28.6 (2.7)	28.6 (1.6)
Control group	16 (4.7)	24 (4)	26.7 (3.6)	28.6 (2.7)	29.7 (0.5)
pH					
Hemoperfusion group	7.3 (0.05)	7.4 (0.08)	7.4 (0.08)	7.5 (0.04)	7.5 (0.05)
Control group	7.2 (0.1)*	7.3 (0.12)	7.4 (0.04)	7.4 (0.04)	7.4 (0.06)
Hemodynamic data, mean (SD)					
MAP (mmHg)					
Hemoperfusion group	74.7 (8.4)	76.6 (14)	78.5 (11.2)	80.2 (15)	78.3 (11.7)
Control group	78.8 (13.4)	83.3 (15)	90.3 (9.5)	87.8 (14.4)	78.7 (10)
Noradrenaline (µg/kg/min)					
Hemoperfusion group	0.8 (0.5)	0.7 (0.7)	0.5 (0.6)	0.3 (0.7)	0.4 (0.8)
Control group	1.3 (0.7)	1.1 (1.2)	0.3 (0.6)	0.2 (0.4)	0.2 (0.3)
Dobutamine (µg/kg/min)					
Hemoperfusion group	1.5 (4.6)	3.2 (5.4)	3.1 (4.6)	2 (4.5)	2.1 (4.5)
Control group	0.5 (1.6)	–*	1.6 (4)	1.6 (4)	0.8 (2.0)
Central venous oxygen Saturat					
Hemoperfusion group	72 (6.5)	62 (12)	61,4 (5.5)	63.5 (8.6)	64.1 (15)
Control group	66 (8)	61 (8.6)	65.2 (7.7)	57.3 (9.9)	58.4 (5.3)
Cardiac index (L/min/m^2^)					
Hemoperfusion group	3.3 (0.76)	2.6 (0.6)	3.2 (0.9)	3.1 (0.9)	3.8 (1.2)
Control group	3.3 (1.18)	2.5 (0.6)	2.6 (0.7)	2.7 (0.8)	2.5 (0.5)
ISVR (dynes/cm^−5^/m^2^)					
Hemoperfusion group	1113.6 (430)	1468.8 (751)	1115.4 (420)	1097 (522)	1261 (699)
Control group	1689.9 (881)	2745.5 (1719)	2528.5 (585)*	2427 (483)*	2600 (400)
EVLWi (ml/kg)					
Hemoperfusion group	7.5 (3)	6.9 (2.7)	6.3 (1.8)	7 (2.7)	7.5 (4)
Control group	7.7 (3.5)	6.3 (1.8)	6.1 (1.7)	5.7 (1.9)	–
PVPi					
Hemoperfusion group	2.1 (0.6)	1.8 (0.7)	1.8 (0.57)	1.9 (0.7)	1.75 (0.75)
Control group	1.6 (0.1)	1.6 (0.2)	1.7 (0.3)	1.3 (0.2)	–
GEDVi					
Hemoperfusion group	950 (380	965 (324)	872 (277)	832 (196)	978 (408)
Control group	1000 (392)	761 (348)	1107 (532)	809 (99)	–
Renal data, mean (SD)					
Serum creatinine (mg/dl)					
Hemoperfusion group	3.8 (2)	2.6 (1.2)	1.9 (0.9)	1.6 (0.6)	1.8 (0.8)
Control group	2.9 (1.3)	1.8 (1.2)	1.5 (0.9)	1.5 (1)	1.5 (0.9)
Azotemia (mg/dl)					
Hemoperfusion group	119 (36)	84 (33)	67.5 (28)	67 (28)	79 (37)
Control group	106 (38)	62 (19)	62 (19)	66 (30)	82 (41)

*MAP* mean arterial pressure, *ISVR* indexed systemic vascular resistances, *EVLWi* extravascular lung water index, *PVPi* pulmonary vascular permeability index, *GEDVi* global end-diastolic volume index

* *p* < 0.05

Table 4 reports the evolution of biological parameters in the two groups over the first 5 days. Lactate, C-reactive protein, procalcitonin, NGAL, and suPAR decreased significantly compared to the baseline, but did not differ between groups. Pro- and anti-inflammatory interleukins gradually decreased, but the decrease did not differ between groups.

**Table 4:** Biological results

Patients in hemoperfusion group/control group	Day 1 (*n* = 18) 9/9	Day 2 (posttreatment) (*n* = 16) 9/7	Day 3 (*n* = 15) 9/6	Day 4 (*n* = 15) 9/6	Day 5 (*n* = 15) 9/6
Lactate (mg/dl), mean (SD)					
Hemoperfusion group	56.7 (33)	41.3 (32.7)	35.2 (34)	26.6 (10.4)	21.3 (14.2)
Control group	56.4 (26.7)	37.7 (26.3)	19.8 (7.5)	17 (3.8)	17 (3.8)
CRP (mg/dl), mean (SD)					
Hemoperfusion group	33.5 (10.7)	30.4 (14.9)	27.6 (19.5)	16.8 (9.7)	14.1 (7.8)
Control group	24.6 (10.3)	24.1 (10.3)	12.9 (5)	8.2 (5.7)	7.4 (9.3)
PCT (ng/ml), mean (SD)					
Hemoperfusion group	83 (92)	43 (59)	33.2 (45.8)	19.9 (27.2)	17.7 (23)
Control group	71 (67)	40 (24)	27.9 (21.2)	12.7 (12.4)	6.1 (5.4)
Adren (nmol/l), mean (SD)					
Hemoperfusion group	17.8 (4.8)	10.9 (3.7)	7.2 (2.4)	5.6 (2.1)	6.4 (1.8)
Control group	17.7 (5.8)	12.3 (3.3)	6.7 (1.8)	4.1 (2)	4.8 (3.6)
IL-6 (ng/ml), mean (SD)					
Hemoperfusion group	1115 (777)	387 (548)	317 (400)	94.5 (120)	367.8 (577)
Control group	8302 (6830)*	3698 (6285)	634 (1292)	9.8 (4.3)	19.2 (14.2)
IL-8 (pg/ml), mean (SD)					
Hemoperfusion group	566.8 (291)	271 (278)	144 (88)	141 (118)	150 (126)
Control group	2766 (3684)	1412 (3090)	781 (1254)	29.4 (8.6)	40.6 (57)
IL-10 (pg/ml), mean (SD)					
Hemoperfusion group	427.1 (339)	178.5 (122)	283.8 (287)	162.7 (164)	131.7 (198)
Control group	3411 (8650)	335 (361)	231 (323)	54.4 (47.4)	77 (95)
IL-1β (pg/ml), mean (SD)					
Hemoperfusion group	4.7 (1.8)	3.7 (0.93)	3.6 (0.6)	3.4 (0.4)	5.3 (3)
Control group	35.1 (90)	2.1 (1.5)	89 (195)	2.4 (1.5)	1.8 (1.5)
TNFα (pg/ml), mean (SD)					
Hemoperfusion group	177.2 (111)	56.8 (26.7)	78.2 (108)	47.7 (36.5)	49.1 (51)
Control group	386 (493)	76.6 (72.8)	170 (355)	28.7 (20.7)	27.4 (8)
SuPAR (ng/ml), mean (SD)					
Hemoperfusion group	23.1 (9.8)	24.5 (12)	24.9 (17.5)	21.8 (13.3)	16.1 (6.8)
Control group	34.2 (21.2)	24.8 (9.4)	23.8 (15.3)	18.2 (11.5)	15.3 (5.4)
NGAL (ng/ml), mean (SD)					
Hemoperfusion group	2331 (1028)	1725.2 (645)	1261 (382)	987 (346)	749 (197)
Control group	2264 (1444)	1284 (1037)	783 (671)	574 (593)	600 (674)

*CRP* C-reactive protein, *PCT* procalcitonin, *Adren* adrenomedullin

* *p* < 0.05

**Fig. 1:**
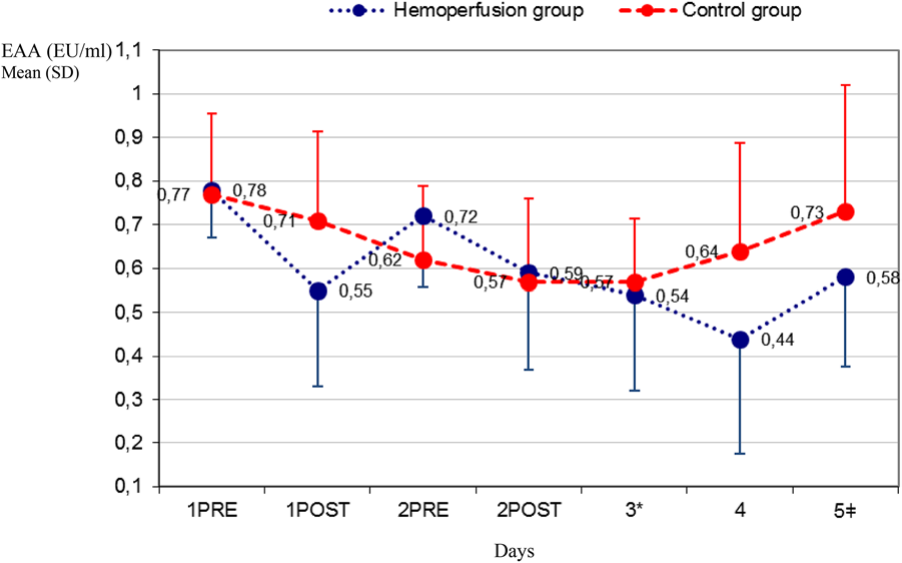
Endotoxin activity levels in the first 5 days. On day 3, EAA had decreased significantly in both groups with respect to the baseline value: hemoperfusion group 0.54 versus 0.78; *p* = 0.02 and control group 0.57 versus 0.77; *p* = 0.05. On day 5, we observe a significantly decreased EAA with respect to the baseline value in the hemoperfusion group 0.58 versus 0.78; *p* = 0.03

Figure 1 shows the evolution of endotoxin plasma activity in the two groups from day 1 to day 5. At baseline, both groups had similar, elevated EAA values. On day 3, EAA had decreased significantly in both groups with respect to the baseline value, but on day 5, the decrease was significant only in the hemoperfusion group.

## Discussion

This prospective study analyzed the clinical and biological effects of hemoperfusion with polymyxin B combined with CRRT in a homogenous population of patients with endotoxic septic shock and multiorgan failure who underwent invasive hemodynamic monitoring. We found that hemoperfusion with polymyxin decreased plasma endotoxin activity, but did not significantly improve clinical or biological parameters.

Many studies (mainly from Japan) have reported that hemoperfusion with polymyxin B decreased endotoxin levels [12–15, 17, 22]. Two large randomized trials, EUPHAS [18] and ABDOMIX [19], tested hemoperfusion with polymyxin B in critical patients, but did not analyze plasma endotoxin levels. In our study, where elevated plasma endotoxin was an inclusion criterion, patients who received hemoperfusion with polymyxin B had significantly decreased endotoxin levels at day 5 compared with the control group.

Unlike other studies, we found no improvement in multiorgan dysfunction, mortality, or biomarkers in patients who underwent hemoperfusion with polymyxin B compared to the control group. Various observational studies showed diverse benefits from hemoperfusion treatment, including improvement in respiratory and hemodynamic parameters, as well as increased survival. In contrast to Vincent et al. [16], who found improved cardiac and renal function, we found no differences in the improvement in organ failure between the hemoperfusion and control groups.

In a systematic review, Cruz et al. [17] concluded that hemoperfusion treatment was associated with improvements in mean arterial pressure, use of vasoactive drugs, Pa02/Fi02 ratio, and mortality. By contrast, the ABDOMIX study [19] and Iwagami et al.’s retrospective study [31] found no improvements. The EUPHRATES trial [21], a multicenter, placebo-controlled, blind trial in 50 ICUs in the USA and Canada, randomized patients with septic shock and EAA > 0.6 to treatment with or without two sessions of hemoperfusion with polymyxin B. Although the initial results showed no reduction in overall mortality at day 28 in the treated group versus the control group, a post hoc analysis limited to the 244 patients with EAA between 0.6 and 0.9 (115 treated patients vs. 129 controls) found a significant reduction in absolute mortality (26.1% in the treated group vs. 36.8% in controls) and a relative mortality reduction of 30%. Romaschin et al. [32] suggest that current polymyxin B filters are probably ineffective in patients with EAA > 0.9. In our study, all patients had initial EAA between 0.6 and 0.9, and none had EAA > 0.9 at inclusion.

After the publication of these trials in 2017, two systematic reviews were published. In the first, Chang et al. [33] found that polymyxin B hemoperfusion reduced mortality in selected patients with intermediate and high risk of disease severity. In the second, Fujii et al. [34] concluded that the treatment does not decrease mortality or the number of organ failures and should therefore not be used routinely.

Our study selected patients who met stringent inclusion criteria (septic shock probably due to gram-negative bacteria with elevated endotoxemia and multiorgan failure) and received homogeneous treatment, including CRRT. The mortality in our study (38.9%) is close to the range reported for similar patients in other studies. Microbiology studies confirmed that infections were predominantly due to gram-negative microorganisms. All patients received appropriate empirical treatment with broad-spectrum antibiotics and early source control. The same procedures were carried out in all patients who received hemoperfusion with polymyxin B, and no complications of this treatment were observed. Our failure to find clinical improvements with hemoperfusion with polymyxin B is likely due to the small sample size limiting the statistical power. Furthermore, concomitant CRRT might mask the effect of hemoperfusion with polymyxin B.

A systematic review of 41 recent articles dealing with the removal of cytokines with extracorporeal techniques found that standard hemofiltration was generally poor at removing cytokines and that high cut-off hemofiltration techniques with large-pore filters were consistently better [35]. Although the technique used in our study was standard hemofiltration, we found a significant decrease in cytokines in both groups, probably derived from the global effect of the treatment administered. In 2009, Payen et al. [6], analyzing several cytokines (IL-6, IL-1ra, and MCP-1) in patients with septic shock randomized to early CRRT or no CRRT, found no differences between the two groups regarding the decrease in cytokines. Analyzing cytokines in patients in the ABDOMIX study, Coudroy et al. [36] found no differences between patients treated with hemoperfusion with polymyxin B and controls. Thus, it seems likely that the decrease seen in both groups in our study was due to the overall effects of treatment rather than to CRRT alone.

Although CRRT techniques have long been used in the treatment of septic shock, the best modality, dose, and time to start remain unclear. In our study, in which 72% of patients had baseline RIFLE scores of Failure, CRRT consisted of hemofiltration at a dose of 35 ml/kg/h and was started within the first 24 h of septic shock. Recently, the ELAIN [37] and AKIKI [5] studies found differences in survival in relation to whether CRRT was initiated early or late. Another recent study found very early onset was associated with poorer outcome due to incorrect dosing of antibiotics [38] and side effects. Studies testing high dialysis flows (> 50 ml/kg/h) in septic patients have failed to find improvements in outcomes [4, 6, 7], and the Kidney Disease: Improving Global Outcomes guidelines [39] recommend prescribing an effluent volume of 30–35 ml/kg/h to achieve a flow of 20–25 ml/kg/h. Within the overall management of septic patients, it is difficult to discern the specific effects of CRRT on outcomes.

To our knowledge, no published studies have prospectively compared septic patients undergoing CRRT and polymyxin B hemoperfusion versus patients undergoing CRRT alone. CRRT might influence the effects of polymyxin B hemoperfusion, thus making it difficult to find significant differences between the two groups. Moreover, the reduction in mortality achieved with improvements in the overall management of septic patients also makes it more difficult to find relevant differences related to specific treatments.

Our study has some important limitations. Patients were not randomized to the hemoperfusion and control groups. Nevertheless, to minimize the selection bias, we included all patients consecutively according to stringent inclusion criteria and applied homogeneous treatment protocols; moreover, the demographic, clinical, and hemodynamic parameters were similar in the two groups. Another limitation is that the inclusion criterion requiring patients to need CRRT might have delayed the initiation of hemoperfusion. Starting hemoperfusion treatment alone would have allowed earlier initiation of hemoperfusion, as in the recently EUPHRATES study [21], where the mean time to starting hemoperfusion was 4 h. However, when our study was designed, our ethics committee deemed the evidence supporting early hemoperfusion insufficient. Nevertheless, our inclusion criterion for CRRT was a RIFLE score of Injury or worse, and 88% of patients started CRRT (with or without hemoperfusion) within 24 h of ICU admission, which is similar to the inclusion criteria of the EUPHAS study [18]. Finally, the early death of three patients in the control group resulted in missing values that could have affected some of our results (mortality, length of mechanical ventilation and CRRT, values of endotoxin and cytokines).

Most studies published on the treatment with polymyxin B do not analyze endotoxin activity levels, and those that do only do not present a control group to compare. In this study, we analyzed the EAA in both groups, which does not measure the absolute value of plasma endotoxins. Clearly, endotoxin levels decreased faster in patients who received hemoperfusion with polymyxin B than in those treated with CRRT alone. Since the filter used for CRRT (AN69) has a very low endotoxin adsorption capacity, we can infer that the decrease in endotoxin in the hemoperfusion group was a consequence of the polymyxin B cartridge. On the other hand, although the hemoperfusion group had lower EAA values than the control group on day 3, the two values are similar. These findings are very similar to those of the recent EUPHRATES study, probably because the EAA is an inadequate reflection of the absolute endotoxin value.

Taken together with the results of recently published trials, our results suggest that further studies are necessary to clarify the efficacy of hemoperfusion with polymyxin B, especially in patients with elevated blood endotoxin level and multiorgan failure.

## Conclusions

Hemoperfusion with polymyxin B decreases blood endotoxin levels, although we found no improvement in clinical and biological parameters. Further studies in larger samples of specific patient populations are necessary to assess the efficacy of polymyxin B hemoperfusion.

## Additional file

**Additional file 1.** Biomarkers analysis methods.

## Abbreviations

EAA: Endotoxin activity assay
CRRT: Continuous renal replacement therapy
CRP: C-reactive protein
PCT: Procalcitonin
AKI: Acute kidney injury
RIFLE: Risk, Injury, Failure, Loss of Kidney Function, and End-Stage Kidney Disease
APACHE: Acute Physiology and Chronic Health Evaluation
SOFA: Sequential Organ Failure Assessment
NGAL: Neutrophil gelatinase-associated lipocalin
SuPAR: Soluble urokinase-type plasminogen activator receptor
