# Supplementary material for: Impact of hemoperfusion with polymyxin B added to hemofiltration in patients with endotoxic shock: a case–control study
Source: Ann Intensive Care. 2018 Dec 7;8:121. doi: 10.1186/s13613-018-0465-8 (PMC6286296; doi:10.1186/s13613-018-0465-8)
Supplement: Supplementary file 1 — Additional file 1. Biomarkers analysis methods. [file 13613_2018_465_MOESM1_ESM.docx]

**Supplementary material**

To measure neutrophil gelatinase-associated lipocalin (NGAL), we used an enzyme-linked immunosorbent assay (ELISA) according to the manufacturer’s instructions (BioPorto Diagnostics A/S, Denmark) on plasma samples diluted 1/100 in microwells coated with a monoclonal antibody to capture human NGAL. The kit uses a horseradish peroxidase (HRP)-conjugated monoclonal antibody to detect bound NGAL, which is incubated with a color-forming substrate and measured in a microplate reader at 650 nm. The limit of detection was 0.008 ng/mL, and the results were expressed in ng/ml.

To measure soluble urokinase plasminogen activator receptor (suPAR), we used a simplified double monoclonal antibody sandwich ELISA with monoclonal antibodies against human suPAR according to the manufacturer’s instructions (suPARnostic kit, BioPorto Diagnostics A/S, Denmark) on plasma samples diluted 1/10. The absorbance at 450 nm was measured using a microplate reader. The limit of detection was 0.1 ng/mL, and the results were expressed in ng/ml.

To measure IL10, IL1-beta, IL-6, IL-8, and TNF-alpha, we used a bead-based multiplex immunoassay according to the manufacturer’s instructions (MILLIPLEX® MAP multiplex biomarker panel; Millipore, Spain) on plasma samples. The results were expressed in pg/ml.
